# Diagnostic Challenges and Management of Fibromyalgia

**DOI:** 10.7759/cureus.18692

**Published:** 2021-10-11

**Authors:** Aniqa G Qureshi, Saurav K Jha, John Iskander, Chaithanya Avanthika, Sharan Jhaveri, Vithi Hitendra Patel, Bhuvana Rasagna Potini, Ahmad Talha Azam

**Affiliations:** 1 Medicine and Surgery, Jinggangshan Medical University, Jian, CHN; 2 Internal Medicine, Kankai Hospital, Birtamode, NPL; 3 Family Medicine, American University of Antigua, St. John's, ATG; 4 Medicine and Surgery, Karnataka Institute of Medical Sciences, Hubli, IND; 5 Pediatrics, Karnataka Institute of Medical Sciences, Hubli, IND; 6 Medicine, Smt Nathiba Hargovandas Lakhmichand Municipal Medical College (NHLMMC), Ahmedabad, IND; 7 Family Medicine, GMERS Medical College and Hospital, Valsad, IND; 8 Internal Medicine, Gujarat Cancer Society Medical College and Research Center, Ahmedabad, IND; 9 Internal Medicine, Kamineni Institute of Medical Sciences, Hyderabad, IND; 10 Internal Medicine, Allama Iqbal Medical College, Lahore, PAK

**Keywords:** diagnostic difficulties, serum biomarkers, chronic inflammation of skin, fibromyalgia and cytokines, fibromyalgia and inflammation

## Abstract

The World Health Organization regards chronic pain to be a public health concern. In clinical medicine, fibromyalgia (FM) is the most prevalent chronic widespread pain disease. In terms of impairment, consumption of health and social resources, and impact on primary and speciality care systems, it has reached worrisome proportions. This disease is frequently managed by primary care providers. Because of its intricacy, fibromyalgia diagnosis and treatment can be difficult.

Fibromyalgia is a controversial condition. It might appear ill-defined in comparison to other pain conditions, with no clear knowledge of pathophysiology and hence no particular targeted therapy. This invariably sparks debates and challenges. There is no obvious cut-off point that distinguishes FM from non-FM. The diagnosis of fibromyalgia has been complicated by several factors, including patients' health-seeking behaviour, symptom identification, and physician labelling of the disease.

Fibromyalgia is currently considered a centralized pain condition, according to research that has improved our understanding of its etiopathology. A multidisciplinary strategy combining pharmacological and non-pharmacological therapies based on a biopsychosocial paradigm can result in effective therapy. Cultural and psychosocial variables appear to be a recent development in fibromyalgia, and they appear to have a larger influence on physician diagnosis than severe symptom levels in FM patients. Although physicians rely on FM criteria as the only way to classify FM patients in research and clinical settings, some crucial elements of the diagnostic challenge of fibromyalgia remain unsolved - invalidation, psychosocial variables, and diverse illness manifestation are some examples.

Beyond the existing constructional scores, physicians' judgment gained in real communicative contexts with patients, appears to be the only dependable route for a more accurate diagnosis for fibromyalgia. We have performed an exhaustive review of the literature using the keywords "Fibromyalgia", "challenges" and "diagnosis" in PubMed and Google Scholar indexes up to September 2021. This article aims to examine the causes, diagnosis, and current treatment protocols of FM, as well as discuss some continuing debates and diagnostic challenges which physicians face in accurately diagnosing fibromyalgia.

## Introduction and background

Fibromyalgia (FM) is among the most impairing causes of chronic widespread musculoskeletal pain. Insomnia, fatigue, cognitive problems, anxiety, and depression are the other characteristic manifestations [1-3]. The worldwide prevalence of FM ranges from 0.2% to 6.6%, with a female preponderance of 3:1 [4-6]. Although it can affect people of any age [7], FM is most commonly diagnosed in women of 30-35 years [8].

FM has been known for several decades; however, the diagnosis remains a challenge owing to the lack of a specific pathophysiological explanation [9,10]. The changes seen from 1990 to 2016 in the diagnostic criteria suggested by the American College of Rheumatology (ACR) have helped; however, it still heavily depends on clinical findings like multisite pain index and somatic symptom severity. Consequently, they miss a large population of patients [11]. Furthermore, many clinicians do not fully comply with the criteria [12]. According to a survey by Wolfe et al., the average time for a patient to get diagnosed with FM is 2.3 years after first presenting to the health setup, and the number of physicians visited in the meantime is 3.7 [13]. This delay is most likely due to the unspecific clinical features and the lack of clinicians’ comprehension of FM [14]. Patients suffering from FM are found to have marked physical limitations and poor overall quality of living and the delayed diagnosis adds to their frustration [15-17].

The biggest diagnostic challenge for FM has been the absence of a reliable objective measure of disease activity [18]. Without specific diagnostic tests, underdiagnosis and late diagnosis remain challenges to be dealt with [13,19]. The misdiagnosis further translates to failed treatment regimens and a high financial burden on the individual and the system [20,21]. Hence, the future of FM diagnosis relies on finding a valid biomarker [22,23].

At present, there is a lack of strong evidence-based guidelines for the treatment of fibromyalgia. The general approach is a mix of patient education, cognitive behavioural therapy, exercise, and pharmacological therapy [24-26].

We have comprehensively reviewed available literature about the pathogenesis, current diagnostic methods, factors associated with delayed diagnosis, diagnostic challenges, possible future of diagnosis, and treatment options of FM in this article.

## Review

Pathogenesis of fibromyalgia

Despite extensive efforts to define the origin and progress of FM over the past three decades, the complete etiopathogenesis remains uncertain. Several factors like genetic predisposition, environmental exposure, hormonal factors, and neural factors have been implicated in triggering FM; however, there is no conclusive evidence yet [27,28]. Our literature review found that currently, there are three main hypotheses for the pathogenesis of FM [29-31]. We have discussed each of them briefly.

A. Central Sensitization

At present, the concept of FM being a “centralized sensitivity syndrome,” originally put forward by Yunus et al., is the most accepted one [29]. According to this hypothesis, alteration in the pain pathway in the central nervous system results in hyperalgesia, allodynia, enhanced temporal summation, and hypersensitivity to various external stimuli like sounds, touch, and lights seen in FM patients [32].

Many studies have substantiated the concept of central sensitization by revealing a lack of balance between the nociceptive and the anti-nociceptive systems in FM patients [33]. Functional imaging studies of the brain of FM patients have shown higher than usual activation and distorted connectivity in areas of the brain controlling pain [34,35]. Also, there is evidence of less grey matter in brain areas like the cingulate cortex, frontal orbit, and insula, which regulate painful signals [36]. The cerebrospinal fluid (CSF) analysis of FM patients further strengthens the hypothesis with findings of increased levels of neurotransmitters (like substance P, Calcitonin gene-related peptide, vasoactive intestinal peptide) that facilitate pain. Furthermore, the levels of neurotransmitters involved with pain attenuation like noradrenaline, serotonin, and dopamine are decreased in FM patients compared to healthy subjects [37].

FM patients are also believed to have a cognitive-emotional sensitization to pain, which explains the impaired responses (hypervigilance, catastrophizing, and avoidance) to painful or other adverse stimuli. According to this concept, the working of the pain network in the central nervous system is influenced by cognitive factors [38-40]. The higher prevalence of psychiatric diseases like depression in patients of FM supports this assumption. However, it is still unclear whether the cognitive changes start the central sensitization to pain or the long-term pain leads to mood changes [41].

The role of chronic sensitization in decreasing the threshold for pain has already been described extensively in disorders like myofascial pain syndrome, irritable bowel syndrome, and interstitial cystitis. However, it has not thoroughly explained the other characteristic features of FM like fatigue and sleep disturbances [42].

B. Dysautonomia-Related Neuropathic Pain Syndrome

FM is viewed as stress-related dysautonomia by some researchers. They propose that psychological stress, physical trauma, different types of infections, or other stressors lead to uninhibited sympathetic hyperactivity in susceptible individuals with the maladaptive autonomic nervous system. This concept is verified by sympathetic hypo-reactivity (low heart rate variability) to orthostatic stress on diverse heart rate studies in FM patients [43-45]. Moreover, it better explains the FM pain, which is experienced in the absence of an external stimulus, and the accompanying allodynia and paresthesia. It also explains the other common symptoms of FM like non-restorative sleep, fatigue (a ceiling effect of sympathetic hyperactivity), and anxiety [46,47].

According to this hypothesis, changes in the dorsal root ganglia are responsible for sympathetically triggered pain. Typically, dorsal root ganglia have few sympathetic fibres, but in FM, stressors-mediated nerve growth factor overexpression induces sympathetic nerve proliferation. Consequently, there is excessive sensory neuron firing. Mutation in the sodium voltage-gated channel alpha subunit 9 (SCN9A) gene produces a defective Nav 1.7 sodium channel in the dorsal root ganglia leading to the severe neuropathic pain seen in FM. These sodium channels situated in the dorsal root ganglia are the gatekeepers of pain transmission from peripheral receptors. In total, nine such sodium channel subunits (Nav 1.1-Nav 1.9) have been found distributed throughout the peripheral and central nervous systems [48-50]. Thus, drugs targeting the mutated sodium channel can be a prospect for FM treatment [30].

The concept of FM as a dysautonomia-associated neuropathic pain syndrome has been reinforced by the recent reporting of small fibre neuropathy (SFN) in a large population of fibromyalgia patients [51]. It is a type of neuropathy that affects the small somatic and autonomic nerve fibres leading to sensory and autonomic dysfunction. SFN affects nerve fibres in a distal-to-proximal fashion resulting in various clinical features like paresthesia, numbness, allodynia, and hyperalgesia. SFN has previously been reported in diseases like diabetic neuropathy and vitamin B12 deficiency [51-53]. A study by Oaklander et al. has reported the presence of SFN in 41% of fibromyalgia patients compared to 3% seen among normal controls. Either skin biopsy or noninvasive corneal confocal microscopy can diagnose SFN. These objective tests can be helpful to overcome the diagnostic challenges of FM [54].

C. Inflammatory Syndrome

According to this hypothesis, immune system activation plays a crucial role in the increased excitability of the pain pathways [55]. There is evidence of increased levels of pro-inflammatory markers like interleukins (IL) 1, 6 and 8, tumour necrosis factor (TNF), neuropeptide Y, corticotropin-releasing hormone, leptin, and substance P in the body fluids of FM patients [56-58]. It is suggested that stressors can upregulate the inflammatory mediators, which stimulate the glial cells and thus culminate in peripheral as well as central neuroinflammation [59,60].

The results of various studies are contradicting, with some demonstrating no difference in inflammatory markers level among FM patients and healthy controls [61]. In addition, studies comparing the erythrocyte sedimentation rate or C-reactive protein between FM patients and healthy controls have also shown conflicting results [27,62,63].

The proponents of the inflammatory theory suggest elevated cytokine levels, distinct cytokine/chemokine profile, elevated neutrophil-lymphocyte ratio (NLR), and elevated platelet counts can be helpful diagnostic markers of FM. Anti-inflammatory drugs are also candidates for FM treatment [64].

Diagnostic protocols

Over the years the diagnosis of fibromyalgia has undergone several changes. The term fibromyalgia syndrome came into use in the 1990s by the American College of Rheumatology (ACR) disposing of the previously used term fibrositis. At the time, to attain a diagnosis, it was necessary to elicit pain on palpation, seen in 18 body points bilaterally, by applying pressure of up to 4 kilograms/square centimetre (4kg/cm^2^). In addition, a three-month history of generalized pain in the axial skeleton and at least three-quarters of the body quadrants was needed. After the 1990s a connection arose between fibromyalgia and neurobiological findings. With more interest in the ACR criteria, it led to numerous studies revealing many co-diagnoses such as irritable bowel syndrome and chronic fatigue syndrome [10].

Although the ACR criteria of 1990 did not include commonly prevalent symptoms and required a tender point exam (applying pressure of up to 4kg/cm2) which was unrealistic it still played a prominent role in setting the base criteria for diagnosing fibromyalgia [65]. Up until 2010, the diagnosis of fibromyalgia was based only on a complete clinical evaluation, it relied mainly on the criteria of widespread pain, of at least three consecutive months of pain and tenderness with palpation [33].

Since 2010, new ACR criteria have been presented and are based on two new components: the score on the Symptom Severity Scale (SSS) score (range 0-12) and the Widespread Pain Index (WPI) (range 0-19) [2]. Also, the 2011 fibromyalgia survey diagnostic criteria were fulfilled if the following three circumstances are established: (1) the WPI ≥7 and the SSS score ≥5, or WPI is 3-6 plus SSS score ≥9; (2) For three months symptoms have been present; and (3) the patient's symptoms aren’t otherwise explained by another disease process. [66].

After the publication of the 2010/2011 criteria, the criteria progressed from a chiefly chronic pain disorder to a multi-symptom disorder while also removing the tender point exam as a prerequisite for diagnosis. In 2016, the preceding precedent was revisited to attain a more accurate diagnostic criterion of fibromyalgia. To achieve the 2016 amended criteria, the patient must meet the following, generalized pain characterized in at least four of five regions and present for at least three months and a WPI >7/19 and SSS score >5/12, or also acceptable is a WPI of 4-6/19 and SSS score ≥9/12. In approaching the WPI the patient demonstrates the number of painful regions that they have experienced in the past seven days ranging from 0-19 including right/left lower and upper leg, buttocks/hip, lower and upper arm, shoulder, and jaw. It also includes the abdomen, lower and upper back, and neck.

The SSS plays a role in that it's based on the average of how drastic a patient experiences cognitive impairment (concentration and memory), unrefreshing sleep and fatigue in the past seven days, the scoring ranges from (0-3) with 0 being "no problem" and 3 being "severe problem." The SSS also considers symptoms that the patient has been experiencing in the past six months. Symptoms include depression, pain in the lower abdomen, and headaches, and this scoring is based on whether it is present or not (0-1) [67]. Overall, the SSS ranges from 0 to 12 and the polysymptomatic distress scale is a combination of the SSS and the WPI ranging from 0-31 [68].

Diagnostic challenges of fibromyalgia

Fibromyalgia diagnosis has been one of the challenges because many patients are unable to describe their presenting complaints and sometimes doctors do not identify the patient's chronic pain as fibromyalgia pain. Some physicians consider a diagnosis of fibromyalgia will affect the impact on patient health so physicians must counsel the patient in such a way that improves patient compliance. According to a US national health interview survey, 75% of patients reported that they are not satisfied with a physician diagnosis of fibromyalgia [69]. Accurate and timely diagnosis can improve the health outcomes of fibromyalgia patients. Studies have revealed that delayed diagnosis has resulted in worse outcomes for the patients [70].

ACR Criteria for Diagnosis

The American College of Rheumatology formulated the diagnostic criteria for fibromyalgia that is tenderness. According to a study, many people have tenderness in the full body while others don't feel symptoms of pain. A survey was also conducted to investigate fibromyalgia diagnostic complications, patients presenting complaints were recorded and no need for tender examination. If the patient tender points were 11/18 then the patient is suffering from severe FM [70].

Multifocal Pain

In patients, fibromyalgia could be suspected if they have multifocal pain but no experience of injury and dominant features of musculoskeletal pain. Major commonly observed symptoms are headache, pelvic pain, sore throat, abdominal pain, and chest pain. So these multifocal pain affects the diagnosis process and further complicates this process [70].

Non-applicable Results of Laboratory Testing

Laboratory testing has not been very applicable in the diagnosis measurement of fibromyalgia but mainly tests include C-reactive protein, thyrotropin, complete blood count, vitamin-D, serum chemistries, and erythrocyte sedimentation. Fibromyalgia can be easily confused with many other diseases, leading to difficulty in diagnosis. Lack of specific investigation has been one of the most critical factors that contribute towards the delayed diagnosis of fibromyalgia [71]. Mostly, doctors have to rely on the patient's symptoms to make an accurate diagnosis. Lack of specific investigation often leads to patients undergoing multiple investigations to eliminate other possible causes of their debilitating symptoms [72]. One of the greatest challenges for medical providers while making a diagnosis of fibromyalgia is to avoid over-investigation to prevent potential iatrogenic harm to the patients [73].

Comorbidities and Stigmatization of the Disorder

Challenges in diagnosis include multiple comorbidities, symptoms, and stigmatization of disorder that may interfere with a fibromyalgia diagnosis. Other challenges include patient non-compliance and lack of treatment adherence. Another reason is the sceptical role of society to delay diagnosis. According to the National Fibromyalgia Association (NFA) survey report, accurate diagnosis of fibromyalgia takes optimum five years and disease progression takes place due to inadequate management. 

Other complications include emotional distress, maladaptive behaviour, poor and psychiatric ailments that lead towards negative consequences [72].

Heterogenous Condition and Phenotypical Changes

Fibromyalgia is a heterogeneous condition; phenotypes change with time and symptoms appear include physical exhaustion, sleep issues, weight fluctuations, and cognitive difficulties, weakness, swelling in extremities, and heat cold intolerance [71,73]. Its boundaries are fuzzy and linked with other complications so it's a challenge to diagnose it.

Functional Disorders

Many functional disorders are also associated with fibromyalgia such as heartburn, chest pain, palpitations, and pelvic complaints. Such functional disorders further complicate the diagnosis of fibromyalgia, leading to misinterpretation. In females, comorbid diagnosis includes endometriosis, dysmenorrhea, vulvar vestibulitis, interstitial cystitis, and vulvodynia. In males, non-bacterial prostatitis could be observed so these overlapping of diseases make it difficult to diagnose fibromyalgia [71].

Dermatological Associations

In fibromyalgia, some skin problems have similarities with systemic lupus erythematosus (SLE) as it involves Raynaud-like, malar flushing, livedo reticularis, and reddening of hands so these are misdiagnosed as SLE [71]. This causes further confusion in accurately diagnosing fibromyalgia.

Considering It as a Diagnosis of Exclusion

Fibromyalgia is mostly considered as a diagnosis of exclusion. Doctors rule out all other causes of pain, fatigue, mood disturbances, and sleep-related disorders before considering fibromyalgia as a potential cause of the patient's symptoms. It accounts for a significant delay until the clinician reaches a point where fibromyalgia is considered a possible cause of the patient's symptoms. Proper education of the clinicians can significantly reduce the time taken to diagnose this condition [74].

Invalidation of Patient’s Symptoms

It has also been observed that invalidation of the patient's symptoms by their medical provider has also been one of the reasons for delayed diagnosis. Patients have to face this issue of invalidation with their medical providers, families, and relatives. This impacts a patient's quality of life and becomes a hurdle in the early diagnosis of a patient [13].

Lack of Knowledge Among Clinicians

Lack of proper awareness and knowledge also contribute toward a delayed diagnosis of fibromyalgia. According to the study by Choy et al. (2010), 45% of physicians had no idea about ACR fibromyalgia classification criteria. Educating doctors about the diagnosis and management of patients with fibromyalgia can go a long way in improving the time taken to diagnose this condition [13].

Recent studies have found an association between delayed diagnosis of fibromyalgia and worse response to different treatment options. It implies that quicker diagnosis and treatment may result in better health outcomes and quality of life for fibromyalgia patients [75].

Treatment options

Fibromyalgia syndrome (FMS) management requires an adequate diagnosis and a symptom-based approach to treatment. With fibromyalgia being under the radar due to limited knowledge regarding the disease and its pathogenesis, the development of disease-modifying therapy is a challenge many physicians encounter. Managing fibromyalgia based on evidence-based treatment plans is another one of the challenges faced by many physicians. The treatment of fibromyalgia patients requires an interdisciplinary approach instead of a focussed pharmacological treatment. It includes education of the patient, along with pharmacological therapy and alternative medicine techniques [76]. Patient education regarding the diagnosis and available treatment options increases the chances of treatment adherence. The inclusion of cognitive behavioural therapy (CBT) to enhance effective coping with the disease and promote self-efficacy is recommended as a part of all treatment plans by most physicians [77]. American Pain Society (APS) and European League Against Rheumatism (EULAR) criteria do not consider any evidence-based findings but are established based on literature searches from recent clinical trials [78].

Strategies to Manage Fibromyalgia

For the accurate diagnosis of the disorder and to formulate a treatment plan, patient education, physician education, and appropriate goal setting are vital. Physicians need to be provided with the required tools and training to recognize the symptoms. Being on par with ongoing clinical trials and updates regarding the latest treatment options available and educating the patient regarding their availability, and assisting them in making a decision should be the goal of physician education.

There is improved diagnostic accuracy and a delay in the initiation of treatment with the development, validation, and implementation of tools to simplify symptom assessment [79]. 

Supporting patients in understanding and accepting the disease and educating them regarding self-management can increase adherence to treatment in the long run. Knowledge regarding the current limitations in the availability of treatment options and engaging the patients in formulating a treatment plan increases the likelihood of positive outcomes.

Pharmacotherapy

APS and EULAR put forward many evidence-based treatment guidelines for fibromyalgia [80]. All these studies recommend a common pharmacologic approach to the treatment of FMS, which include four broad drug classes- 1) serotonin-norepinephrine reuptake inhibitors (SNRIs), 2) selective serotonin reuptake inhibitors (SSRIs), 3) tricyclic anti-depressants (TCAs), and 4) anti-epileptic drugs (AEDs) [80]. The Food and Drug Administration (FDA) approved duloxetine, pregabalin, and milnacipran for the treatment of fibromyalgia [24]. These three drugs have shown similar potency in alleviating pain but their potentiality to manage other symptoms differs considerably. Their different pharmacodynamic and safety profiles often make one of the drugs a better initial choice than the others for an individual patient [25].

According to an internet survey, the most commonly used medications for treating fibromyalgia were acetaminophen, ibuprofen, naproxen, and amitriptyline. Some of the top 10 drugs that were rated as most helpful were hydrocodone, alprazolam, oxycodone, diazepam, and zolpidem. There is a discrepancy between the most commonly used and the most effective medications according to the survey. This may be associated with the heavy use of over-the-counter drugs, as they are generally cheaper than prescription drugs [26].

Neuromodulatory Drugs

Tricyclic anti-depressants (TCAs): The prototype of this class, amitriptyline is a well-studied and evaluated drug. It aids in improving pain, sleep disturbance, fatigue and improves the daily quality of life. The typical dose is 10-50 milligram (mg) daily. A meta-analysis was done using amitriptyline, milnacipran, and duloxetine for the treatment of fibromyalgia, which indicated that amitriptyline could improve fatigue, pain, and quality of life in fibromyalgia patients and was found superior to duloxetine and milnacipran [81]. Common side effects include dry mouth, constipation, sedation, confusion, orthostasis, urinary retention, weight gain, sexual dysfunction.

Serotonin and norepinephrine reuptake inhibitors (SNRIs): The FDA approved duloxetine and milnacipran for the treatment of fibromyalgia. They improve pain and aid patients with depressive symptoms. A recent review of milnacipran for adult fibromyalgia stated that it is effective only in about 40% of patients, providing moderate pain relief. Effective dosages were 100-200 mg [82]. Adverse effects include nausea, dry mouth, constipation, drowsiness, hyperhidrosis, and decreased appetite.

Anti-epileptic drugs (AED): Pregabalin and gabapentin are anti-epileptic drugs used in the treatment of fibromyalgia. Pregabalin is one of the first drugs to be approved by the FDA in 2007 for fibromyalgia [83]. Although these drugs were the prototype for fibromyalgia treatment in the past, they are not effective in symptom treatment of fibromyalgia in the judgment of recent reviews and trials [84]. Blurred vision, dry mouth, drowsiness, oedema, dizziness, weight gain, difficulty with concentration and attention are some of the common adverse effects of these drugs.

Analgesics

The use of opioids in fibromyalgia is controversial, and the guidelines vary in their recommendations.

Opioids have relatively high abuse potential, and it is essential to avoid such medications. There is no evidence-based data support for the use of opiates in fibromyalgia. And the recent evidence of opiate-induced hyperalgesia also suggests their limited usefulness [85,86]. In a survey, hydrocodone plus acetaminophen is found to be more helpful in alleviating the symptoms than oxycodone plus acetaminophen [87]. Moreover, people come to the physicians with the expectation of getting pain medications so that they can get relief. Physician hesitation due to abuse potential and prior drug use/substance use history along with Drug Enforcement Administration (DEA) and prescription program regulations make it difficult to prescribe such medications. Therefore, the frustration of patients doesn't allow for an efficient physician-patient relationship to form. 

Most or almost none of the currently available drugs are fully effective against the entire spectrum of fibromyalgia symptoms, namely pain, fatigue, sleep disturbances, and depression [88].

Newer Drugs

Transdermal testosterone is a novel agent for the treatment of fibromyalgia and appears to reduce pain response in animal models. The rationale behind this is the presence of aromatase-positive cells in the primary pain processing site in the spinal cord, that is dorsal root ganglion, where transmission of pain information to the thalamus and cerebral cortex originates [89]. In fibromyalgia patients, especially women, a decreased testosterone level increases substance P and induces wind up [90].

A co-agonist of glutamate at the N-methyl-D-aspartate receptors (NMDA), "NYX-2925", was studied and found to be safe and well-tolerated in healthy volunteers, the results are in favour of its continued clinical development for the treatment of chronic pain conditions [91].

Other anti-depressants like mirtazapine, a pre-synaptic alpha-2 antagonist, esreboxetine (norepinephrine reuptake inhibitor), and desvenlafaxine (serotonin-norepinephrine reuptake inhibitor) are currently being explored in clinical trials.

Probiotics are also among the proposed treatments for fibromyalgia. They were thought to act via the gut-microbiota-brain axis but they did not exhibit any promising results apart from improved decision-making capabilities with no effect on cognition, quality of life, pain, depressive, and anxiety symptoms [90].

Non-pharmacological Modalities

The guidelines for encompassing non-pharmacological modalities in the treatment of fibromyalgia vary, and a multidisciplinary approach that combines pharmacotherapy, cognitive therapy, physical therapy, and natural remedies is favoured. The non-pharmacological management of fibromyalgia has a significant impact on the clinical manifestations, symptoms, and quality of life in comparison to pharmacologic treatment. The various forms of non-pharmacological modalities include exercise regimens, diet, behavioural therapies, complementary and alternative medicinal practices that play a role in the overall management of the symptoms of a fibromyalgia patient. A holistic model of treatment is required in individuals with more severe disease and for those who fail to respond to initial treatment [92]. Limited availability of pharmacological treatment options is a reason for considering alternative medicine.

Diet and Exercise

Some studies have reported that dietary changes can be efficacious and have positive effects on pain. Nutrients that reduce neuronal inflammation and improve muscle strength are deficient in some patients with fibromyalgia. Pain and functional symptoms in FMS patients improved with the incorporation of various diets like the low-calorie diet, a vegetarian diet, or a low fermentable oligosaccharides, disaccharides, monosaccharides, and polyols (FODMAPs) diet. Diet impacted some patient-reported outcomes, such as quality of life, quality of sleep, anxiety, depression, and inflammatory biomarkers also showed an improvement with these interventions [93].

Exercise is an effective component of treatment that improves pain and quality of life and decreases the burden of the disease. Aerobic exercises, strength training, and flexibility training have been shown to provide benefits in fibromyalgia [94]. Exercise is easier to incorporate into the daily routine. Starting initially with low-intensity exercises and gradually improving to 30-60 mins of activity at least three times a week. In a 2007 meta-analysis to evaluate the effectiveness of different exercise modalities, aerobic exercise improved global well-being, physical functionality, and pain [95]. Resistance training is associated with decreased depression, and muscle strengthening exercises increase the quality of life.

Dancing is a type of aerobic exercise that can be used in fibromyalgia as an alternative therapy. Belly dancing is another form of physical activity found to be efficacious in improving functional capacity, pain, quality of life, and improving the body image of women with fibromyalgia syndrome [96]. According to a recent study, three months of treatment of patients with FMS with Zumba dancing as an alternative treatment was also found to be effective in improving pain and daily functioning [97].

Alternative Therapies

Acupuncture: It is a traditional Chinese medicine practice where thin needles are inserted at different points on the body. It is generally in use to relieve pain. In fibromyalgia, the effect of acupuncture correlates with a change in serum serotonin levels [98]. Pain and stiffness of fibromyalgia improve with acupuncture. It works by reducing inflammation, causing the release of endorphins, and creating a calmer mind [99]. Many studies agree with its usefulness in fibromyalgia compared to no treatment, but the effects do not last after six months [100]. Tai chi, an internal Chinese martial art practised for defence training, health benefits, and meditation improved fibromyalgia symptoms [101].

Electrical therapy: Fibromyalgia, apart from pain, can present with anxiety, depression, fatigue, decreased working memory, and attention referred to as "fibro fog". Transcranial electric and magnetic stimulation is a non-invasive brain stimulation method effective in the modulation of brain areas and their perception of pain. Therapeutic electrical stimulation via transcutaneous electric nerve stimulation appears to have a potential role in the treatment of pain in FMS [102].

Thermal therapy: Body warming and cryotherapy have a minor role in the management of fibromyalgia. Modulation of nociception can be accomplished through the application of both hot and cold temperatures. They act on the opioid pain inhibitory system and alternation of rhythm in temperature [103]. It has anti-inflammatory and analgesic properties. According to an internet survey, patients tried heat therapy with significant improvement in the symptoms [104]. In a study involving patients undergoing mud-bathing daily for a month, pain sensitization improved, and a decrease in serum biomarkers such as triglycerides and C-reactive protein was observed [105]. 

Mind and body therapy, vibroacoustic and rhythm therapy, and massage therapy are other alternative ways that are in use for the management of fibromyalgia [106,107].

Due to the complex nature of fibromyalgia, many patients are prescribed multiple medications to manage the various symptoms and accompanying comorbid medical and psychiatric disorders. Although polypharmacy can be an effective clinical strategy for patients with complex medical conditions, several potential problems and adverse effects are to be considered. The use of multiple medications can exacerbate adverse events or an increased risk of unwanted drug-drug interactions. Effective communication between patients and treatment providers may be the most effective way to manage the potential risks of multiple medication use [108].

Due to the chronicity of the disease, one of the main concerns in the treatment of fibromyalgia patients is poor compliance with long-term treatment. Physicians should be able to tailor the therapy to the most prevalent manifestations in a patient. Internet-based platforms and group educational programs can aid in providing additional social support. Increasing treatment adherence and improving the quality of life is an added benefit.

Biomarkers: possible future of fibromyalgia diagnosis

Fibromyalgia is a disease that does not have universal criteria for a diagnosis due to a wide range of possible clinical examinations. Several findings suggest the validity of genetics, environmental factors such as trauma, illness, stress-response, sleep patterns, in FM progression [26]. The progress made in studying the pathogenesis of fibromyalgia could help change the diagnosis of FM from a subjective perspective to an objective one. Even though chances of these biomarkers being employed clinically are thin on the ground due to practical reasons such as financial burden, lack of specificity, they do help identify correlated disorders and eventually help in making a better diagnosis.

Genetic Outlook

In an investigation of the genome, the first-degree relatives of individuals with fibromyalgia displayed a 13.6-fold greater risk of developing the disease [109]. The association of genetic variants and pain response was further strengthened by the fact that the family members of FM cases were more likely to suffer from other chronic pain conditions such as irritable bowel syndrome, temporo mandibular joint (TMJ) disorder, and headaches [110] (Table 1). Hence conducted studies provide insight into candidate genes as a plausible marker for FM diagnosis. 

**Table 1:** The list of candidate genes and their key single nucleotide polymorphism (SNPs) predisposing to fibromyalgia (FM) and other clinical correlations. The objective abnormalities found in FM and other pain-related conditions also showed particular gene polymorphism.

CANDIDATE GENE	VARIANTS/ POLYMORPHISM	REMARKS	ASSOCIATED SNPs
5-hydroxytryptamine (5-HTT)	S/S genotype	Was more prevalent in FM with depression/ anxiety compared to only FM patients [111].	
Catechol-O-Methyl Tranferase (COMT)	Low activity (Met/Met)	Was predominant in FM patients and had exacerbated symptoms of pain, fatigue, stress, and sleep disturbances [112,113].	
	Low activity (Val/Met)	Was also frequently associated with anxiety, depression, and disability in FM women [114,115].	rs4680
SLC64A4 (serotonin transporter gene)	On chromosome 17p11.2-q11.2	Which were linked with chronic pain disorders like TMJ [116].	5-HTTLPR ( Serotonin Transporter Linked Promoter Region) [110]
HTR2A (serotonin receptor)		Seen repeatedly in FM onset [117].	rs6313
Transient Receptor Potential Vanilloid Channel 2 (TRPV2)	Expressed in mechano-thermo-responsive neurons in the dorsal and trigeminal ganglia [118]	Might cause impaired pain threshold in FM cases [109].	
Trace Amine Associated Receptor 1 (TAAR1)	Mediates impaired dopamine availability	Associated with increased pain sensitivity, the hallmark of FM patients [118].	rs8192619, rs4129256 [119]
RGS4 (G protein signal 4 regulator gene)	Locus coeruleus, in the dorsal horn of the spinal cord, and the bed nuclei of the stria terminalis [119]	Responsible for decreasing inhibition of pain perception [109].	rs10799897, rs2842003, rs2805050 [119]
Dopamine D4 receptor	Decreased frequency of 7-repeat allele	Frequently found in association with FM patients [120].	
µ1 opioid receptor	Lower frequency of the 118 G allele	Found in patients of FM [121].	
CNR1 (cannabinoid receptor 1 gene)	Encoding Cannabinoid Receptor 1 (CB-1) variants	Was associated with migraine, Irritable Bowel Syndrome along with FM [122,123].	rs6454674, rs1078602, rs10485171 [119]

Epigenetic Outlook

Prior research has shown the effects of early life experience and environmental factors on genome function and the phenotype without changing the DNA sequence through the mechanism of epigenetics [124]. The long-term changes in the central and peripheral nervous systems seen in chronic pain have been mediated by epigenetic pathways like change in the state of methylation, histone modification, and the expression of micro ribonucleic acids (miRNAs) [125].

Deoxyribonucleic Acid (DNA) Methylation

The first study investigating epigenetic changes in FM women focused on a genome-wide methylation pattern. The study highlighted 69 differentially methylated sites in cases against controls and 91% of these sites were accountable for an increased micronuclei frequency in FM women [126]. The study pointed out the genes mapped on differently methylated sites, indicating the possible involvement of nervous system development, skeletal/organ system development, cell signalling pathways, and chromatin acetylation-deacetylation in FM [127]. A strong correlation of lower DNA methylation level in the promoter region of higher TRPA1 gene expression, particularly in peripheral nociceptors, and gate pain-related responses corresponded to a higher pain threshold [128]. Another research showed an altered methylation level in peripheral blood of FM patients during cortical excitability parameters measurement of both hemispheres [129]. DNA methylation has the potential of a budding biological marker in the diagnosis of FM due to promising results seen in several studies.

Micro Ribonucleic Acids (miRNAs)

MiRNAs are small non-coding ribonucleic acid molecules that regulate the expression of genes. They were found to play a vital role in chronic pain diseases [130] (Table 2). In a profile of miRNA by Bjersing et al., nine miRNAs in cerebrospinal fluid and eight miRNAs in serum were differently expressed in FM cases [131,132]. Masotti et al. also investigated miRNA profiles in the serum and saliva of FM patients and found six miRNAs to be associated with FM [133]. Additional studies are required to reinforce these preliminary findings in larger cohorts to prove the involvement of miRNA in FM pathogenesis as well as aid in diagnosis [109].

**Table 2:** During post transcription of messenger RNA (mRNA), miRNA dysregulation contributes to certain manifestations. The table lists a few miRNAs that were differentially expressed in FM patients (CSF/serum/saliva) in comparison to a healthy population. CSF: cerebrospinal fluid; FM: fibromyalgia

miRNAs	Regulation in FM	Sample type	Associated symptoms
miR-145-5p [131]	Down	CSF	Pain and fatigue
miR-103a-3p let-7a-5p8 [132]	Down	Serum	Sleep quality and pain
miR-374b-5p [132]	Down	Serum	Pain threshold
miR-23a-3p [133]	Down	Serum Saliva	Maintenance of skeletal muscle integrity

Gene Expression

Jones et al. conducted a genome-wide expression profiling in the peripheral blood of 70 patients with fibromyalgia and 70 healthy controls, 421 differentially expressed genes were identified, several were related to pain processing pathways. The test captured a subset of 10 probe sets with a sensitivity of 95% and a specificity of 96% for FM, but it needs to be validated in larger cohorts of patients [134]. Dolcino et al. in a recent study on gene expression profiles in peripheral blood mononuclear cells on 10 patients and 10 healthy subjects showed two out of 298 long non-coding ribonucleic acids (lncRNAs) targeted the most common genes in FM. These studies showcase the role of genetics, epigenetics, and autoimmunity in FM pathology and future diagnosis [135].

Mu-Opioid Receptor on B Lymphocytes

The role of Mu opioid receptor on B lymphocytes in chronic pain disease was studied on three groups of females (FM patients, osteoarthritis (OA) patients, and healthy individuals) and the results showed that the percentage of Mu-positive B cells was statistically lower in FM and OA patients compared to pain-free subjects [136]. Approach to this area could play a pivotal role in the diagnosis of many chronic pain diseases.

Serological markers

The idea of having a simple blood test to diagnose FM seems appealing. But even after many years of research on markers such as inflammatory cytokines, neurotransmitters, and autoantibodies, we do not have any confirmed tests available.

Inflammatory Cytokines

During exercise, muscles release IL-6, which in turn causes secretion of IL-10 and IL-1β (anti-inflammatory cytokines) [137]. IL-10 modulates substance P expression and thereby increases the pain threshold. This study along with the role of light exercise in FM treatment points to a likely subject that could be used as a diagnostic tool.

Neurotransmitters

The levels of neuropeptides Y were frequently higher in FM patients when compared to healthy subjects [138,139]. Substance P and neurokinins are known to regulate pain but substance P’s effect on disturbed sleep was also confirmed in a study [140]. Hence, the possible linkage of substance P with the symptom of disturbed sleep in FM could be made.

Autoantibodies

Extensive studies have been done by many researchers displaying a major correlation between thyroid autoimmunity and FM [141,142]. Immunoglobulin M (IgM) antibodies against phosphatidylinositol were seen in both depression and chronic fatigue syndrome [143]. Hence a likely contender to be considered for diagnosis.

## Conclusions

Fibromyalgia is undeniably a difficult diagnostic and treatment issue. Although the American College of Rheumatology (ACR) has established diagnostic criteria for FM, they are not frequently used in clinical practice. Some health care providers, particularly in basic care are unaware of the condition. In addition to diagnostic challenges, there is a lack of prescription consistency among clinicians. Many patients may not get the right treatment, and those who do are likely to have frequent therapeutic switching or abrupt cessation of therapy. Some patients may have excessive treatment expectations and difficulties coping with their symptoms, which can make treating their illness more challenging.

The varied presentations of FM and ambiguity in understanding its etiopathogenesis and genetic correlations also add to the difficulty in diagnosis. The rapid development of knowledge about the aetiology of pain, inflammation, and behavioural processes, as well as breakthroughs in the field of functional assessment of the brain, should make FM detection and therapy more successful. We encourage further studies and research into improving the currently existing diagnostic protocol for fibromyalgia and we also recommend continued medical education of primary care providers about the diagnosis and treatment of this condition.
